# Learning spin liquids on a honeycomb lattice with artificial neural networks

**DOI:** 10.1038/s41598-021-95523-4

**Published:** 2021-08-17

**Authors:** Chang-Xiao Li, Sheng Yang, Jing-Bo Xu

**Affiliations:** Zhejiang Institute of Modern Physics and Department of Physics, Zhejiang University, Hangzhou, 310027 People’s Republic of China

**Keywords:** Magnetic properties and materials, Quantum information, Computational science

## Abstract

Machine learning methods provide a new perspective on the study of many-body system in condensed matter physics and there is only limited understanding of their representational properties and limitations in quantum spin liquid systems. In this work, we investigate the ability of the machine learning method based on the restricted Boltzmann machine in capturing physical quantities including the ground-state energy, spin-structure factor, magnetization, quantum coherence, and multipartite entanglement in the two-dimensional ferromagnetic spin liquids on a honeycomb lattice. It is found that the restricted Boltzmann machine can encode the many-body wavefunction quite well by reproducing accurate ground-state energy and structure factor. Further investigation on the behavior of multipartite entanglement indicates that the residual entanglement is richer in the gapless phase than the gapped spin-liquid phase, which suggests that the residual entanglement can characterize the spin-liquid phases. Additionally, we confirm the existence of a gapped non-Abelian topological phase in the spin liquids on a honeycomb lattice with a small magnetic field and determine the corresponding phase boundary by recognizing the rapid change of the local magnetization and residual entanglement.

## Introduction

The research on frustrated magnetic systems has attracted broad interest in condensed matter physics over the past several decades due to the strong frustration that can cause various novel quantum phases in these systems, such as quantum spin liquids (QSLs)^1^. QSL is a novel type of quantum state, in which any long-range magnetic order is suppressed by strong quantum fluctuation and is highly entangled even at absolute zero temperature. In addition, this exotic phase of matter is beyond Landau’s phase transition theory and not include any local order parameter or spontaneous symmetry breaking. Specifically, important theoretical insight into QSL physics comes from the study of Kitaev spin liquids (KSLs)^2^, which can be solved exactly through Jordan–Wigner transformation^3,4^. The Kitaev model is highly frustrated and shows a large number of interesting peculiarities, such as long-range entanglement in the ground state and non-Abelian anyon excitation^1^ due to the bond-dependent anisotropic interactions. In particular, motivated by a recent experiment on KSL material candidate: $\alpha$-${{\mathrm{RuCl}}}_3$^5^, one finds that the ferromagnetic (FM) Kitaev model has a field-induced non-Abelian topological phase under an external magnetic field. Then, extensive attention has shifted to the case with a magnetic field^6–9^ and discovered that the topological phase survives only in the presence of a small magnetic field^10^.

Furthermore, when considering complex or realistic systems, analytical methods may fail to extract the relevant physics and numerical approaches, like quantum Monte Carlo simulation^11^ and the density matrix renormalization group algorithm^12^, become necessary. Recently, a new strategy for reducing this complexity is to use machine learning algorithms based on neural networks^13^, such as restricted Boltzmann machines (RBMs)^14^, which is one of the most widely used neural networks in the machine learning community. Modern machine learning technologies can classify, identify or interpret massive data sets such as images, which makes it naturally suitable to analyze the exponentially large information contained in the quantum many-body state. For quantum many-body problems, the commonly interested states, namely the low-lying eigenstates, generally possess an intrinsic structure, obeying the area-law of entanglement^15^. It is noted that the representation power of artificial neural networks when adapted to encode the many-body wavefunctions has been investigated from the entanglement perspective to some extent^16^. Specifically, it was suggested that quantum states represented by RBMs can potentially exhibit a volume-law of entanglement^17^, which indicates a more powerful representation ability than tensor networks. Based on this observation, many researchers utilized machine learning methods to solve strongly correlated quantum systems^18^, simulate the stationary state of open quantum systems^19–22^, and explore the nature of quantum phase transition in many-body systems^23–26^. Despite the rapid developments, the representative features and limitations of neural networks in characterizing the QSL phases remain to be elucidated.

On the other hand, characterizing different phases of matter plays an important role in condensed matter^27^. Unfortunately, it is tough to find characteristics and universal properties of a given phase, like the QSL state, lacking local order parameters. Alternative ways to characterize different phases have recently emerged from the concepts of quantum information science^28^ overcoming this difficulty. Quantum entanglement^15,29,30^ recognized as a key resource was the first and most commonly used one. Besides the developments of bipartite entanglement^31^, which describes only a part of characteristics of many-body quantum systems, multipartite entanglement quantifying entanglement distribution among different parties demonstrates complex structures of quantum states more thoroughly and deserves further investigation. Specifically, a relevant multipartite entanglement, residual entanglement^32^, was recently proposed based on a general monogamy inequality of squared entanglement in an arbitrary *N*-qubit mixed state^33^. Furthermore, quantum coherence is another favored choice for describing quantumness. And it has been applied to the fields of quantum optics^34^, quantum thermodynamics^35^ and quantum algorithms^36^. Recently, a rigorous framework for quantifying quantum coherence^37^ was established, from which arises several good quantifiers like the relative entropy coherence and $l_1$-norm coherence. Different from the traditional approaches used in condensed matter, these quantum information-oriented methods can investigate quantum phases without any knowledge of order parameters and offer new insights for quantum phase transition.

The above discussions may motivate one to consider the following questions: Can neural networks capture the physical natures of QSL phases? In the process of learning QSLs, what are the abilities and limitations of the neural networks? Can the residual entanglement be used to characterize the QSL phases? In this paper, we attempt to show the abilities and limitations of the neural network-based machine learning method to capture QSL states of the spin liquids on a honeycomb lattice with a magnetic field. First, we apply RBMs to learn the ground-state energy and spin-structure factor in the QSL honeycomb lattice, and compare these results with those obtained by exact diagonalization to verify the effectiveness of the neural network-based machine learning method. Then, we investigate the performances of the quantum coherence and residual entanglement via the RBM-based machine learning method, and observe that both of these quantities can be used to determine the boundary between the gapped and gapless QSL phases. It is shown that the residual entanglement is richer in the gapless phase than the gapped spin-liquid phase. Furthermore, we focus on the training of RBMs to learn the physical quantities including the local magnetization and residual entanglement in the QSL honeycomb lattice with a small magnetic field. It is found that both local magnetization and residual entanglement show a steep change near the critical field, which reveals the existence of a non-Abelian topological phase. Our work provides new insights for the machine learning method into the learning of different condensed matter phases, especially the QSLs.

Our paper proceeds as follows. In “Methods”, we briefly introduce the neural network-based machine learning method and outline how to calculate the multipartite entanglement. RBMs are applied to learn physical quantities including the energy, spin-structure factor, quantum coherence and multipartite entanglement for the pure QSL honeycomb lattice and FM QSL honeycomb lattice in “Results”. We finally present conclusion and discussion in “Conclusion and discussion”.

## Methods

In this section, we briefly outline the neural network-based machine learning method and how to calculate the multipartite entanglement.

**Figure 1:**
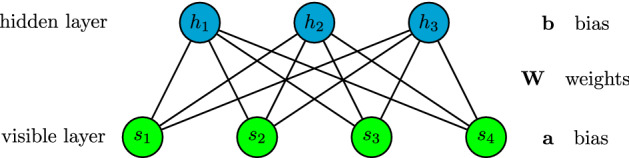
Network diagram of the restricted Boltzmann machine.

To set a general stage for our study, we start with the notion of the restricted Boltzmann machine states^13^. The artificial network structure of RBMs sketched in Fig. 1 is composed of one visible layer with *N* physical spin variables, $s_i=\pm 1$, and one hidden layer with *M* auxiliary spin variables, $h_j=\pm 1$. In this paper, we set the number of hidden units equals to the number of visible ones, namely, $M=N$.

By using the computational basis set, $\left\{ \left| {\vec{s}}\,\right\rangle \right\}$, one can express a general quantum state as

1 $$\begin{aligned} \left| \Psi \right\rangle =\sum _{\left\{ s_i\right\} }\Psi (s_1,\dots ,s_N)\left| s_1,\dots ,s_N\right\rangle , \end{aligned}$$

where $s_i$ is the local quantum number for *i*-th site. An RBM is taken to represent the target many-body quantum state and the coefficient $\Psi ({\vec {s}}\,)$ is encoded as^13^

2 $$\begin{aligned} \Psi (s_1,\dots , s_N) = e^{\sum _i^{N} a_i s_i} \times \Pi _{j=1}^{M} \cosh \left( \sum _i^{N} W_{ij} s_i + b_j \right) . \end{aligned}$$

Here, $a_i$ and $b_j$ are called visible and hidden bias, respectively, and the weights $W_{ij}$ representing the network links are taken to be complex-valued.

In the context of artificial neural networks, the word *learning* means that the parameters of the network, $X=\left\{ {\mathbf{a }}, {\mathbf{b }},{\mathbf{W }}\right\}$ in RBMs, are iteratively optimized to find the minimum of a certain cost function. To train a RBM, we adopt the variational Monte Carlo (VMC) method for optimizing the variational parameters of RBMs.

### Optimization

We initialize all weights ${\mathbf{W }}$ based on a random Gaussian distribution and biases ${\mathbf{a }}$ and ${\mathbf{b }}$ with uniformly sampled numbers in the interval [0, 0.1]. To obtain the ground state of a given Hamiltonian *H*, the expected value of energy $E(X)=\left\langle \Psi |H|\Psi \right\rangle /\left\langle \Psi |\Psi \right\rangle$ is chosen as the cost function, which is a function of the network parameters $X=\left\{ {\mathbf{a }},{\mathbf{b }},{\mathbf{W }}\right\}$. And then the objective of training is to obtain optimal RBM parameters for which the energy function converges to a minimum. To find the global minimum of the cost function, we choose to adopt Adagrad optimizer^38^ in this study. Concretely, the *k*-th network parameter $X_k$ is updated at the *p*-th iteration according to

3 $$\begin{aligned} \begin{aligned} g_k^{p}&=g_k^{\,p-1}+\left( \frac{\partial E^{\,p}(X)}{\partial X_k}\right) ^2\\ X_k^{\,p}&=X^{\,p-1}_k-\frac{\eta }{\sqrt{g_k^{\,p}+\epsilon }} \frac{\partial E^{\,p}\left( X\right) }{\partial X_k}, \end{aligned} \end{aligned}$$

where $\eta$ is the corresponding learning rate, $\epsilon$ is a small cutoff to prevent division by zero, and the process is repeated until the cost function is converged.

### Sampling

However, for the quantum many-body problems, it is hard to optimize such a large number of network parameters in the full Hilbert space. In the stochastic framework, this optimization problem can be solved by using the stochastic reconfiguration^39,40^. During the optimization procedure, the expectation value of a physical observable ${\hat{O}}$ of interest can be written as

4 $$\begin{aligned} \begin{aligned} \langle {\hat{O}}\rangle =\frac{\left\langle \Psi \right| {\hat{O}} \left| \Psi \right\rangle }{\left\langle \Psi |\Psi \right\rangle } =\frac{1}{\sum _{\vec {s}}|\Psi ({\vec {s}})|^2}\sum _{\vec {s}} \,|\Psi ({\vec {s}})|^2\sum _{{\vec {s}} ^{\,\prime }}\frac{\Psi ({\vec {s}}^{\,\prime })}{\Psi ({\vec {s}})} \langle {\vec {s}}\,|{\hat{O}}|{\vec {s}}^{\prime }\rangle =\sum _{\vec {s}}p({\vec {s}}\,)O_{\mathrm{loc}}({\vec {s}}\,), \end{aligned} \end{aligned}$$

where $p({\vec {s}}\,)=|\Psi ({\vec {s}}\,)|^2/Z$ corresponds to a classical probability distribution with $Z=\sum _{{\vec {s}}}|\Psi ({\vec {s}}\,)|^2$ and the local expectation contribution of a certain configuration ${\vec {s}}$ is $O_{\mathrm{loc}}({\vec {s}})=\sum _{{\vec {s}}^{\,\prime }}\frac{\Psi ({\vec {s}}^{\,\prime })}{\Psi ({\vec {s}}\,)} \langle {\vec {s}}\,| {\hat{O}}|{\vec {s}}^{\,\prime }\rangle$. Therefore, the expectation values of observables, such as energy and its gradient, can be estimated at each learning step by using the Markov chain Monte Carlo method^39^ with Metropolis local sampler^41^. The network parameters are updated iteratively until the optimal energy is obtained and then the training ends.

Specially, we present the calculation of the *n*-site reduced density matrix, which is essential in the evaluation of quantum information quantities. Take the simplest case, namely the calculation of the one-site reduced density matrix on *i*-th site, for example, the elements of the reduced density matrix, $\rho _i^{s_is_i'}$, can be estimated one by one by exploiting the formula (4) with ${\hat{O}}=|s_i'\rangle \langle s_i|$. Finally, one can use these estimated elements to reconstruct the required reduced density matrix with enforced Hermitian and normalization condition. The implementation is based on the NETKET library^42^, which is an open-source Python toolbox that supports the message passing interface for distributed and parallel computing.

With the help of the neural network-based machine learning method, we can calculate multipartite entanglement: residual entanglement, a useful entanglement indicator in many-body systems^33^. The definition of residual entanglement is based on a general monogamy inequality of the squared entanglement of formation $E_f^2$ in an arbitrary *N*-qubit system

5 $$\begin{aligned} E_f^2(\rho _{A_1|A_2\dots A_N})-\sum _{i\ne 1}^{N}E_f^2(\rho _{A_1A_i})\ge 0, \end{aligned}$$

where $E_f(\rho _{A_1|A_2\dots A_N})$ represents the entanglement between $A_1$ and the rest of the system, and $E_f(\rho _{A_1A_i})$ stands for the bipartite entanglement of the two-qubit system $A_1A_i$. And the residual entanglement is defined as

6 $$\begin{aligned} \tau \equiv E_f^2(\rho _{A_1|A_2\dots A_N})-\sum _{i\ne 1}^{N}E_f^2(\rho _{A_1A_i}). \end{aligned}$$

Multipartite entanglement written in the above form can be interpreted as the global entanglement that does not store in the bipartite spins. Additionally, the first term in Eq. (6) can be expressed as simple as $E_f(\rho _{A_1|A_2\dots A_N})=S(\rho _{A_1})$ for the pure state case^43^, where *S* represents the von Neumann entropy. Further, there is a one-to-one correspondence between the bipartite entanglement $E_f(\rho _{A_1A_i})$ and concurrence $C(\rho _{A_1A_i})$^44^,

7 $$\begin{aligned} \begin{aligned} E_f(\rho _{A_1A_i})&=H_{\mathrm{bin}}\left( \frac{1+\sqrt{1-C^2(\rho _{A_1A_i}})}{2}\right) ,\\ C(\rho _{A_1A_i})&\equiv \max \left\{ 0, \sqrt{\lambda _1}-\sqrt{\lambda _2} -\sqrt{\lambda _3}-\sqrt{\lambda _4}\right\} , \end{aligned} \end{aligned}$$

where $\lambda _i$ is the eigenvalues of matrix $\rho _{A_1A_i}(\sigma _{A1}^y\otimes \sigma _{A_i}^y)\rho ^*_{A_1A_i}(\sigma _{A1}^y\otimes \sigma _{A_i}^y)$ in decreasing order, and $H_{\mathrm{bin}}$ is the Shannon binary entropy function, $H_{\mathrm{bin}}(x)=-x\log _2(x)-(1-x) \log _2(1-x)$.

## Results

In this section, we employ the machine learning approach based on RBMs to investigate the topological phase for the FM QSL honeycomb lattice, and study various physical quantities to provide a better understanding of the application of machine learning in QSLs.

**Figure 2:**
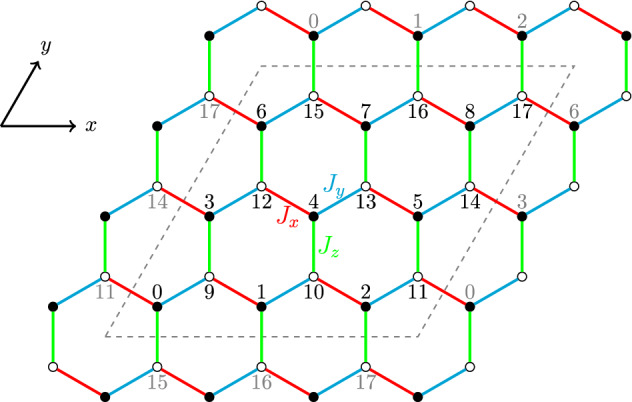
Structure of the Kitaev honeycomb lattice for $N=3\times 3\times 2$.

We consider the FM QSL honeycomb lattice, where a set of localized spin-1/2 particles are placed at a two-dimensional honeycomb lattice with periodic boundary condition (see Fig. 2), subjected to an external magnetic field *h* along the [111] direction. The Hamiltonian is given by

8 $$\begin{aligned} H = -\sum _{\langle i,j \rangle _{\gamma }}J_{\gamma }\sigma _{i}^{\gamma } \sigma _{j}^{\gamma }-h\sum _{i}(\sigma _{i}^x+\sigma _i^y+\sigma _i^z), \end{aligned}$$

where $\sigma _i^\gamma$ represents the Pauli matrix at *i*-th site, $\gamma =x,y,z$ indicates three different nearest-neighbor links of the hexagonal lattice with the coupling interaction $J_\gamma$, and the first term of Eq. (8) is the pure QSL honeycomb lattice exhibiting strongly anisotropic spin exchange couplings. Here, the size of a system is $N=L_x\times L_y\times 2$, where $L_x$ and $L_y$ indicate, respectively, the number of spins along the *x* and *y* directions.

For the case of zero magnetic field $h=0$, the pure QSL honeycomb lattice is known to be solved exactly through a Jordan–Wigner transformation combined with symmetry considerations^4^. There are two kinds of spin-liquid phases^2^ in the parameter space: three gapped ${\mathbb {Z}}_2$ spin liquid phases $A_x, A_y, A_z$ with Abelian anyon excitations for $J_x>0.5, J_y>0.5, J_z>0.5$, respectively, and a gapless phase *B* for other cases, which hosts non-Abelian anyons in the presence of a small magnetic field, for simplicity, we have set $J_x+J_y+J_z=1$ as the energy unit.

**Figure 3:**
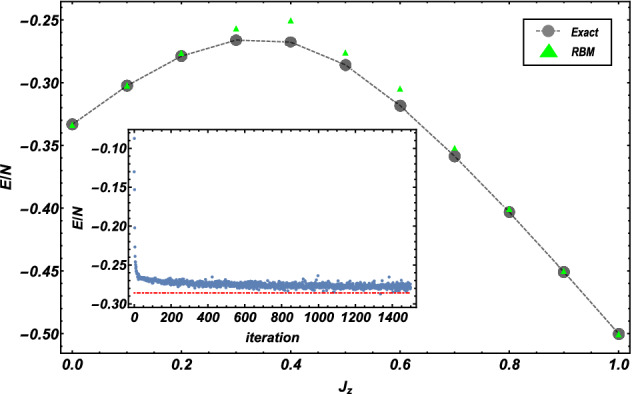
The ground-state energy *E*/*N* computed as a function of the coupling $J_z$ for a $N=3\times 3\times 2$ lattice. RBM (green triangle) and the exact diagonalization (gray solid circle) values are compared. The inset shows the energy expectation value *E*/*N*, at the critical point $J_z=0.5$, as a function of the VMC iterations. The red dashed line in the inset marks the exact value of the ground-state energy. Other parameters: $J_x=J_y=(1-J_z)/2$, $\eta =0.1$, $\epsilon =10^{-7}$.

To assess the effectiveness of RBMs, we apply the RBMs to a pure QSL honeycomb lattice with up to $N=3\times 3\times 2$ sites with periodic boundary condition and optimize the network parameters to learn the ground state. It is found that 1500 iterations are mostly enough for training in this case. The expected values of the ground-state energy for different values of $J_z$ with $J_x=J_y$ obtained by RBM optimization are exhibited in Fig. 3 and compared with the results computed by exact diagonalization. We can see that, for $J_z\lesssim 0.2$ and $J_z \gtrsim 0.8$, a high precision can be reached with a relative error, $\delta =|E_{\mathrm{exact}}-E_{\mathrm{RBM}}|/|E_{\mathrm{exact}}|$, on the order of $\delta \sim 10^{-3}$. We also plot the energy as a function of the iterative steps to visualize the training process at the critical point, $J_z=0.5$, in the inset of Fig. 3, and show an explicit convergence towards to the exact value. In addition, the results of Fig. 3 suggest that RBMs have difficulties learning quantum state in the range around phase transition, and a larger number of network parameters might be required to improve the performance.

**Figure 4:**
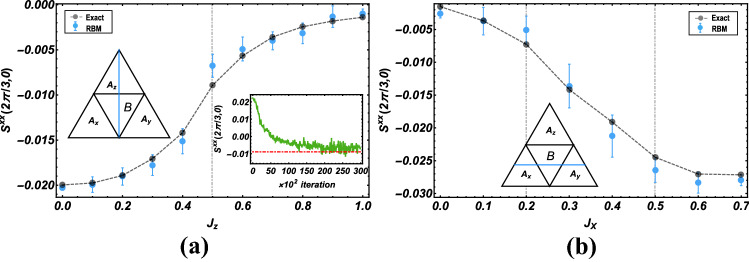
The spin-structure factor $S^{xx}\left( 2\pi /3,0\right)$ (blue solid circle), compared with exact diagonalization results (gray solid circle), as a function of the coupling: (**a**) $J_z$, other parameters are set as $J_x=J_y=(1-J_z)/2$; (**b**) $J_x$, other parameters are set as $J_z=0.3, J_y=0.7-J_x$ for a $N=3\times 3\times 2$ lattice. The blue solid straight lines are plotted to indicate the error bars. Inset in (**a**): the convergence of the structure factor towards the exact value in the training process, at the critical point $J_z=0.5$. The red dashed line in the inset marks the exact value.

Besides the investigation of the energy expectation value, we also consider the ability of RBMs to learn the transverse spin-structure factor,

9 $$\begin{aligned} S^{xx}({\vec {k}})=\frac{1}{N(N-1)}\sum _{l\ne j}e^{-i{\vec {k}}\cdot ({\vec {r}}_{l}-{\vec {r}}_{j})}\left\langle \sigma _l^x \sigma _j^x\right\rangle , \end{aligned}$$

which is experimentally accessible via scattering experiments. In Fig. 4, we display the dependence of the spin-structure factor $S^{xx}(2\pi /3,0)$ on different coupling interactions with the parameters as $J_x=J_y$. It is shown from the inset in Fig. 4a that the structure factor at the critical point, $J_z=0.5$, as a function of the iterative steps. A clear convergence towards to the exact value is found. We also report the structure factor computed for different values of $J_x$ in Fig. 4b, with $J_z=0.3$ and $J_y=0.7-J_x$, where the phase transition is expected to occur at $J_x=0.2$ and $J_x=0.5$. The RBM results of $S^{xx}(2\pi /3,0)$ agree well with the exact solution, especially for the points far away from the critical points.

From the above discussion, we have demonstrated a proof of principle of the RBM-based machine learning method by two successful tests including the ground-state energy and spin-structure factor of the pure QSL honeycomb lattice. It is observed that RBM states at criticality are hard to train because the quantum fluctuation reaches maximum there. In addition, the energy results are more accurate than the spin-structure factor, which means that the convergence of physical quantities lags behind the convergence of the cost function. One possibility to improve the learning of neural networks for capturing more accurate physical quantities is to perform a more precise training to make $\delta$ reach the order of $\sim 10^{-5}$, compatible with the state of the art methods, which may require the involvement of other modern machine learning technologies like deep Boltzmann machines^14^, convolutional neural networks^45^, and recurrent neural networks^46^.

Next, we turn our focus to the performances of the quantum coherence and multipartite entanglement in the pure QSL honeycomb lattice.

**Figure 5:**
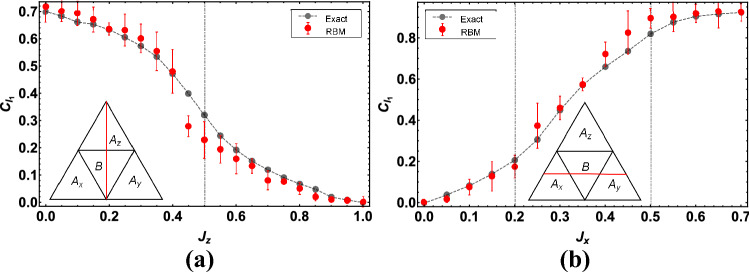
$l_1$-norm coherence $C_{l_1}$ as a function of the coupling strength (**a**) $J_z$ with $J_x=J_y=(1-J_z)/2$, (**b**) $J_x$ with $J_z=0.3, J_y=0.7-J_x$, for a $N=3\times 3 \times 2$ lattice. Gray vertical lines indicate the critical points. The red solid straight lines indicate the error bars.

We train the architecture over $3\times 10^4$ iterations using AdaGrad optimizer and we set the learning rate to $\eta =0.03$, and the cutoff to $\epsilon =10^{-7}$. Here, we adopt the $l_1$-norm quantum coherence^37^, which is defined as the sum of the absolute value of the off-diagonal elements of a density matrix $\rho$, $C_{l_1}\left( \rho \right) =\sum _{i\ne j}\left| \rho _{ij}\right|$. In Fig. 5, we plot the quantum coherence of two nearest-neighbor sites linked by $J_x$ coupling as a function of coupling interactions $J_z$ (Fig. 5a) and $J_x$ (Fig. 5b) for the same parameters as in Fig. 4. The quantum coherence declines with increasing $J_z$ in Fig. 5a, but enhances with increasing $J_x$ in Fig. 5b and changes dramatically near the critical point, agreeing with the previous work^47^ that adopts the relative entropy coherence measure. It is also observed that the value of coherence learned by RBM is pretty close to the exact value, suggesting that the quantum feature of QSL phases is accurately reproduced. Additionally, we also note that for the points near the phase transition, the errors in Fig. 5 are relatively large, and similar behaviors are also observed in the studies of energy and spin-structure factor.

**Figure 6:**
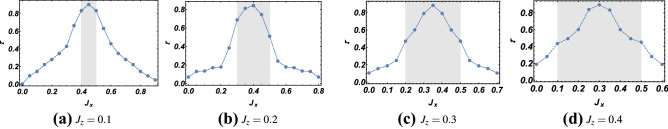
Residual entanglement $\tau$ as a function of coupling interaction $J_x$ with different $J_z$: (**a**) 0.1, (**b**) 0.2, (*c*) 0.3, (*d*) 0.4 for a $N=4\times 4\times 2$ lattice.

Over the past decade, there have been increasing interests in the characterization of quantum phases via entanglement measures. Recent works^48,49^ show that topological quantum phases can be characterized by the behavior of multipartite entanglement using the quantum Fisher information^50^. Additionally, it was also reported that multipartite entanglement in the form of residual entanglement can serve as good indicators to detect quantum phase transitions in several spin models^51^. Hence, it would be worthwhile to study the performance of residual entanglement in the QSL honeycomb lattice to see whether residual entanglement can characterize the QSL physics. As was outlined in “Methods”, we plot the residual entanglement $\tau$ for a $N=4\times 4\times 2$ lattice in Fig. 6 with $J_z=0.1$ (Fig. 6a), 0.2 (Fig. 6b), 0.3 (Fig. 6c), 0.4 (Fig. 6d). It is found that these curves all increase first and then decrease while increasing $J_x$ and approach the maximum at the median value of $J_x$. Interestingly, residual entanglement is richer in the gapless phase *B* than the gapped spin-liquid phases *A*. This suggests that there would be a rapid enhancement of multipartite entanglement as the QSL slides from a gapped region to another gapless one, and residual entanglement can be used to mark out the corresponding transition point.

As mentioned earlier, physical quantities obtained by the machine learning method suffer a small deviation compared with the exact results. In general, this deviation can be reduced by performing a more accurate training and averaging over more samples in Eq. (4). Besides this, however, the one-by-one strategy used in the calculation of density matrix may bring larger accumulative error into the evaluation of quantum coherence and residual entanglement. For this reason, a smarter strategy that can extract the density matrix as a whole would be appealing and we leave it for future studies.

So far, we have confirmed the effectiveness of the RBM method in capturing physical features of the pure QSL honeycomb lattice. Previous theoretical studies found that a small magnetic field along the $\left[ 111\right]$ direction will drive the gapless spin-liquid phase into a gapped topological phase with non-Abelian quasiparticle excitations^2,10^. To better understand the change of the physics for the application of the magnetic field, we apply the RBM method to solve the FM spin liquids on a honeycomb lattice and specifically consider the behavior of the local magnetization and residual entanglement with respect to the external field.

**Figure 7:**
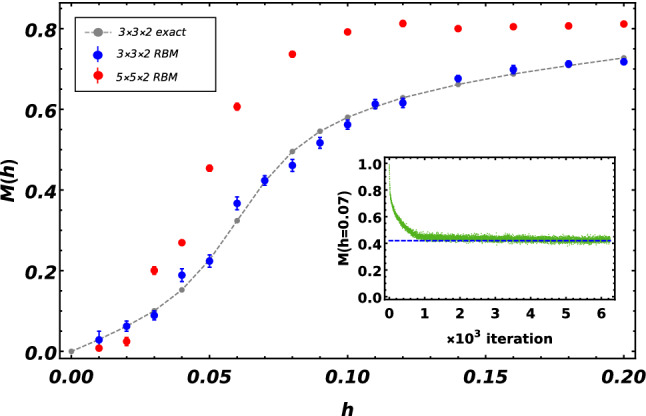
Magnetization *M*(*h*) computed as a function of the magnetic field *h*. Exact values obtained by exact diagonalization (gray solid circle) are compared. The inset shows the convergence of magnetization at $h=0.07$ for $N=3\times 3\times 2$ lattice towards to the exact value (blue dashed line) in the training process. Parameters: $J_x=J_y=J_z=2$.

**Figure 8:**
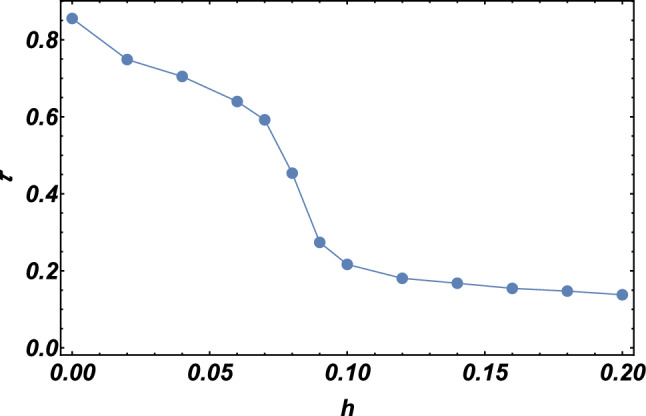
Residual entanglement $\tau$ computed as a function of the magnetic field *h* for a $N=4\times 4\times 2$ lattice. Parameters: $J_x=J_y=J_z=2$.

First, we compute the local magnetization *M* according to

10 $$\begin{aligned} \begin{aligned} M&=\sqrt{\sum \nolimits _{\gamma }(M_{\gamma })^2}\\ M_{\gamma }&=\frac{1}{N}\sum \nolimits _{i=1}^{N} \left\langle \sigma _i^\gamma \right\rangle ,\quad \gamma =x, y, z, \end{aligned} \end{aligned}$$

for the FM QSL honeycomb lattice. According to the size of the system, we train the complete architecture over $10^3$-$10^4$ iterations using the Adagrad optimizer with a learning rate $\eta =10^{-2}$ and a small cutoff $\epsilon =10^{-7}$. The expected values of magnetization *M* obtained by RBM, for $N=3\times 3\times 2$ and $N=5\times 5\times 2$ lattices, are plotted in Fig. 7 as a function of external magnetic field *h* with coupling $J_x=J_y=J_z=2$. It is observed that the local magnetization gains a nonzero value with an arbitrary small magnetic field. Specifically, the gapless QSL phase is driven into a gapped non-Abelian topological phase by a small magnetic field and this topological phase exists only within the interval $0<h\lesssim 0.07$. As the parameter *h* is tuned through the critical value $h^*\approx 0.07$, the local magnetization shows a steep change, which implies the happening of a quantum phase transition. For the region $h>0.07$, the magnetization shows a monotonic increase with respect to *h* and gradually approaches a saturate value, which corresponds to a fully polarized state.Furthermore, these two distinct regions can also be distinguished by investigating the behavior of the residual entanglement with respect to the external magnetic field. We display the residual entanglement $\tau$ as a function of the external field *h* with coupling strength $J_x=J_y=J_z=2$ for a $N=4\times 4\times 2$ lattice in Fig. 8. It is quite clear from Fig. 8 that the residual entanglement first decreases slowly with the increase of magnetic field and then drops rapidly near a critical field that agrees with the one marked by the local magnetization. For lager magnetic field, the system comes into a partial polarized phase with relatively small residual entanglement as expected. Additionally, the residual entanglement stays at a relatively large value within the gapped non-Abelian topological phase, and such a rich entanglement may be advantageous in the topological quantum computation. Interestingly, both the behaviors of the local magnetization and residual entanglement can be used to confirm the existence of the gapped topological phase and determine the phase boundary.

## Conclusion and discussion

In summary, we have explored the abilities and limitations of the RBM-based machine learning method in representing the QSL states and specifically considered the FM QSL honeycomb lattice. Besides, we have provided a table containing different variational energies obtained in Supplementary. We note that a previous study^52^ has examined the capability of RBMs to find ground-state energy of the Kitaev honeycomb model. However, they only focus on the specific parameter choice $J_x=J_y=J_z=1$, and we not only focus on the full phase diagram containing three gapped phases and one gapless phase with $J_x+J_y+J_z=1$ but also consider the effect of the external field. By investigating the accuracy of the learned energy and structure factor in four phases, we confirmed the validity of the machine learning method in solving the QSL honeycomb lattice. Then we investigated the behavior of quantum coherence and residual entanglement and find that these quantities can serve as good indicators for the quantum phase transition between the gapped and gapless spin-liquid phases in the spin liquids on a honeycomb lattice. Furthermore, we recognized the existence of a gapped non-Abelian topological phase, which might be useful in performing the topological quantum computation, by further investigating the local magnetization and residual entanglement for the case with an external field.

Based on the success of the RBM-based machine learning method in the exploration of the FM QSL honeycomb lattice, one can expect that the neural network-based machine learning method might be a good tool to study the physics of general QSL states. As we have mentioned in the main section, however, there are still many difficulties that we have to face to make this method more reliable and powerful, which will be the focus of future research. On the one hand, the effects of finite size are large, therefore, the precise extrapolations to the thermodynamic limit are needed. To this end, a more reliable and powerful technique would be required to perform accurate finite-size analysis of the quantities presented here. On the other hand, we found that the convergence of physical quantities lags behind the convergence of the ground-state energy (see an example of magnetization in Supplementary Fig. S1 online), and the one-by-one strategy used in the calculation of density matrix may bring larger accumulative error into the evaluation of physical quantities, which limits the application of neural networks to explore the physical quantities of large-scale systems. In this context, it would be interesting to see how modern machine learning approaches can help tackle these problems. It is noted that some recent works have employed many advanced machine learning techniques, like the transfer learning^53^, convolutional neural networks^54,55^ and recurrent neural networks^56^, to achieve high accurate learning for solving quantum many-body Hamiltonians. On the other hand, the concepts underlie the modern physics, such as symmetry, locality, entanglement and renormalization group^17,57^, have also be used to understand the representing and learning power of the neural networks. We hope that the combination of these two directions can finally make the machine learning method a regular strategy for exploring the physics of quantum matters, especially the QSL physics.

## Supplementary Information

Supplementary Information.
