# Author Correction: Learning spin liquids on a honeycomb lattice with artificial neural networks

**DOI:** 10.1038/s41598-021-02472-z

**Published:** 2021-11-23

**Authors:** Chang‑Xiao Li, Sheng Yang, Jing‑Bo Xu

**Affiliations:** Zhejiang Institute of Modern Physics and Department of Physics, Zhejiang University, Hangzhou, 310027 People’s Republic of China

Correction to: *Scientific Reports* https://doi.org/10.1038/s41598-021-95523-4, published online: 17 August 2021

This original version of this Article contained an error, as the paper did not discuss the related work of Noormandipour et al. (2021). As a result, Reference 52 was omitted and is listed below,

Noormandipour, M., Sun, Y. & Haghighat, B. Restricted boltzmann machine representation for the groundstate and excited states of kitaev honeycomb model. https://arxiv.org/abs/2003.07280 (2021)

In addition, the text in the Conclusion and discussion,

“By investigating the accuracy of the learned energy and structure factor, we first confirmed the validity of the machine learning method in solving the QSL honeycomb lattice.”

now reads:

“We note that a previous study^52^ has examined the capability of RBMs to find ground-state energy of the Kitaev honeycomb model. However, they only focus on the specific parameter choice $J_{x} = J_{y} = J_{z} = 1$, and we not only focus on the full phase diagram containing three gapped phases and one gapless phase with $J_{x} + J_{y} + J_{z} = 1$ but also consider the effect of the external field. By investigating the accuracy of the learned energy and structure factor in four phases, we confirmed the validity of the machine learning method in solving the QSL honeycomb lattice.”

The original Article has been corrected.

