# Supplementary material for: Learning spin liquids on a honeycomb lattice with artificial neural networks
Source: Sci Rep. 2021 Aug 17;11:16667. doi: 10.1038/s41598-021-95523-4 (PMC8371168; doi:10.1038/s41598-021-95523-4)
Supplement: Supplementary file 1 — Supplementary Information. [file 41598_2021_95523_MOESM1_ESM.pdf]

Supplementary Materials

Learning spin liquids on a honeycomb lattice with artificial neural networks

Chang-Xiao Li[1], Sheng Yang[1], and Jing-Bo Xu[1*]

[1]Zhejiang Institute of Modern Physics and Department of Physics, Zhejiang University, Hangzhou 310027, People's Republic of China

*xujb@zju.edu.cn

Table S1. Comparison of energy per site between RBM used in this paper, Jastrow wave functions, and DMRG results on $4 \times 4 \times 2$ and $5 \times 5 \times 2$ honeycomb lattices with the external field $h = 0.1$.

4×4	Method	Jz=0.0	Jz=0.2	Jz=0.4	Jz=0.6	Jz=0.8	Jz=1.0	CPU time per iteration	N(iteration)
Energy	RBM	-0.4388(2)	-0.3942(4)	-0.3833(7)	-0.4216(4)	-0.5133(1)	-0.6097(1)	0.2345(s)	1000
	Jastrow	-0.4040(7)	-0.3510(5)	-0.3267(9)	-0.345(2)	-0.4117(5)	-0.5031(3)	0.05(s)	1000
	DMRG	-0.43881	-0.39465	-0.38718	-0.43567	-0.51596	-0.60985	385(s)	50

5×5	Method	Jz=0.0	Jz=0.2	Jz=0.4	Jz=0.6	Jz=0.8	Jz=1.0	CPU time per iteration	N(iteration)
Energy	RBM	-0.4398(3)	-0.3934(3)	-0.3844(4)	-0.4327(5)	-0.5137(1)	-0.6016(1)	0.5955(s)	1500
	DMRG	-0.43536	-0.39141	-0.38383	-0.43523	-0.51595	-0.60314	992(s)	70

1 Computer configuration: Intel® Core™ i5-9600K CPU @ 3.70GHz × 6, Mesa Intel® UHD Graphics 630 (CFL GT2), and 15.5 GB Memory.

2 Truncation error: 10^{-7} for a $4 \times 4 \times 2$ lattice with bond dimension $D = 200$; 10^{-4} for the spin-liquid phase B, 10^{-7} for phase A on a $5 \times 5 \times 2$ lattice with bond dimension $D = 400$.

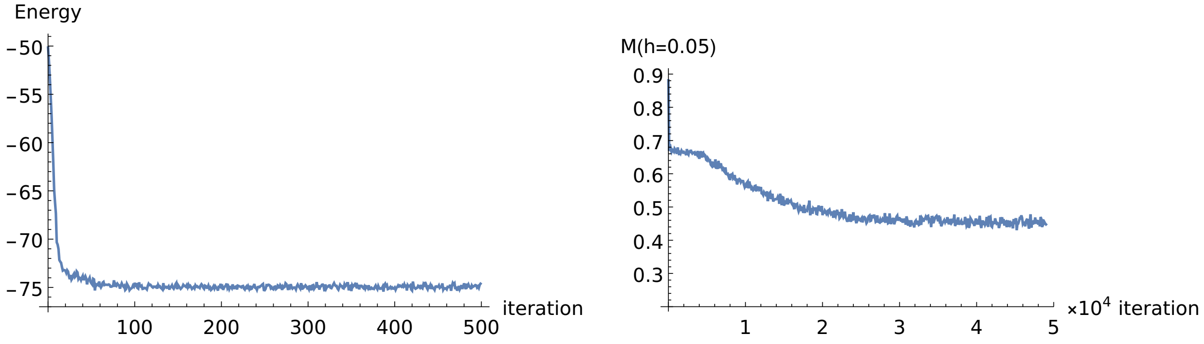

(a) (b)

Figure S1. The convergences of (a) the energy and (b) magnetization in the training process with external field $h = 0.05$, $J_x = J_y = J_z = 2$, for a $N = 5 \times 5 \times 2$ lattice.
